# COSMIC-2 RFI Prediction Model Based on CNN-BiLSTM-Attention for Interference Detection and Location

**DOI:** 10.3390/s24237745

**Published:** 2024-12-04

**Authors:** Cheng-Long Song, Rui-Min Jin, Chao Han, Dan-Dan Wang, Ya-Ping Guo, Xiang Cui, Xiao-Ni Wang, Pei-Rui Bai, Wei-Min Zhen

**Affiliations:** 1 School of Electronic Information Engineering, Shandong University of Science and Technology, Qingdao 266590, China; songccclong@sdust.edu.cn (C.-L.S.); wangdandan@sdust.edu.cn (D.-D.W.); gyp2018@sdust.edu.cn (Y.-P.G.); bprbjd@163.com (P.-R.B.); 2 China Research Institute of Radiowave Propagation, Qingdao 266107, China; jinrm@crirp.ac.cn (R.-M.J.); cuix@crirp.ac.cn (X.C.); zhenwm@crirp.ac.cn (W.-M.Z.); 3 School of Electronic Engineering, Xidian University, Xi’an 710071, China; 4 School of Information Science and Technology, Qingdao University of Science and Technology, Qingdao 266061, China; fs645@qust.edu.cn

**Keywords:** GNSS radio frequency interference, COSMIC-2, GNSS RO, CNN-BiLSTM-Attention, deep learning

## Abstract

As the application of the Global Navigation Satellite System (GNSS) continues to expand, its stability and safety issues are receiving more and more attention, especially the interference problem. Interference reduces the signal reception quality of ground terminals and may even lead to the paralysis of GNSS function in severe cases. In recent years, Low Earth Orbit (LEO) satellites have been highly emphasized for their unique advantages in GNSS interference detection, and related commercial and academic activities have increased rapidly. In this context, based on the signal-to-noise ratio (SNR) and radio-frequency interference (RFI) measurements data from COSMIC-2 satellites, this paper explores a method of predicting RFI measurements using SNR correlation variations in different GNSS signal channels for application to the detection and localization of civil terrestrial GNSS interference signals. Research shows that the SNR in different GNSS signal channels shows a correlated change under the influence of RFI. To this end, a CNN-BiLSTM-Attention model combining a convolutional neural network (CNN), bi-directional long and short-term memory network (BiLSTM), and attention mechanism is proposed in this paper, and the model takes the multi-channel SNR time series of the GNSS as the input and outputs the maximum measured value of RFI in the multi-channels. The experimental results show that compared with the traditional band-pass filtering inter-correlation method and other deep learning models, the model in this paper has a root mean square error (RMSE), mean absolute error (MAE), and correlation coefficient (R^2^) of 1.0185, 1.8567, and 0.9693, respectively, in RFI prediction, which demonstrates a higher RFI detection accuracy and a wide range of rough localization capabilities, showing significant competitiveness. Since the correlation changes in the SNR can be processed to decouple the signal strength, this model is also suitable for future GNSS-RO missions (such as COSMIC-1, CHAMP, GRACE, and Spire) for which no RFI measurements have yet been made.

## 1. Introduction

The GNSS is an essential infrastructure for positioning, navigation, and timing in modern society [1]. It is widely used in many fields, such as intelligent transportation systems, precision measurement, critical infrastructure protection, and space environment monitoring [2,3]. The diverse applications of the GNSS have become the primary support for various mission-critical tasks [4].

However, the signal power the satellite navigation receiver receives is meager, mainly due to the considerable distance between the signal source and the receiver and the complex transmission path [5,6], resulting in the navigation signal being very weak when it reaches the ground. According to the GPS ICD interface document, the minimum received RF signal strength is −158.5 dBW [7]. The meager input signal power makes the GNSS receiver extremely vulnerable to external interference, which, in severe cases, can cause the navigation system to fail. For example, a ground-based GNSS jammer with an Effective Isotropic Radiated Power (EIRP) of only 10 W can be received by an LEO satellite orbiting at an altitude of 1000 km [8]. In practical applications, the power of interference signals is often much higher than 10 W [9], posing a more significant threat to GNSS signal reception.

GNSS radio occultation (RO) satellites are equipped with GNSS receivers that receive electromagnetic signals emitted by high-orbit GNSS satellites. During transmission, radio waves are affected by the Earth’s atmosphere and ionosphere, refracting the signal path and carrying information about the atmosphere and ionosphere. This information can be extracted using scientific inversion techniques [10,11,12]. When the onboard GNSS receiver receives the radio-frequency interference (RFI) signal, the power spectral density of the interference signal is often higher than the noise level. The interference source can be detected and located using spectrum, Doppler, time difference of arrival (TDOA), frequency difference of arrival (FDOA), and other methods [13,14,15,16,17,18]. GNSS interference detection and location based on near-Earth orbit have recently proven feasible. For example, Ashkan Kalantari et al. [19] proposed a single-satellite positioning method based on the frequency-of-arrival (FOA) technique, which can locate an unknown interference source based on the measurements of a single satellite. Murrian et al. [20,21] used the FOTON receiver for long-term Doppler-based interference analysis. Zachary Clements et al. [22] used a two-step positioning method of TDOA and FDOA to locate some real-world interference and spoofing signals on two STRATOS satellites provided by Spire Global Inc. Existing LEO constellation systems such as Hawkeye360 and Spire Global also provide RFI source location services using similar techniques [23,24,25].

Many satellite-borne GNSS data have been collected through international GNSS Radio Occultation missions, such as COSMIC-1/2, MetOp A/B/C, FY3C/D, and GRACE [26,27,28,29]. These data cover multiple aspects, including the atmosphere, ionosphere, and interference signals. However, due to constraints such as transmission bandwidth, open-source data often do not include raw, intermediate frequency (IF) data, making detecting interference based on power spectral density challenging. Despite this limitation, the 1 Hz SNR data included in Level 1b Near Real-Time (NRT) data [30] provide an alternative way to locate interference sources roughly through noise power density analysis. For example, Bonnedal M [31] and Isoz O [32] conducted a statistical analysis of the global distribution of interference signals between 2007 and 2010 using data from the MetOp satellite. Additionally, T. Maximillian Roberts et al. [33] identified the time and locations of interference from 2007 to 2016 based on observed changes in the SNR. However, deep learning techniques have been less frequently applied in existing methods, primarily due to insufficient interference cases to create large-scale training datasets.This situation has improved with the update of RFI measurements in the COSMIC-2 level product in 2022 [34], which can be used as interference labels to promote the application of deep learning in satellite-based interference detection and location. So far, only Cosmic-2 has released RFI measurements, while most GNSS-RO missions have released SNR data. A deep learning model trained with data from existing RFI labels can be used to detect RFI in other GNSS-RO and GNSS-R missions that do not have RFI measurements but have SNR data. At this point, a rough location of the RFI can be determined by projecting the satellite’s orbital position at the time of the RFI detection to the ground.

This paper discusses the application of deep learning technology in detecting abnormal GNSS receiver SNR data from GNSS RO satellites. RFI events are identified through anomaly detection, and the position of the RFI source is estimated by the orbital coordinates when the SNR is abnormal. The second part describes the mechanism by which RFI affects the SNR of the COSMIC -2 GNSS receiver; the third part describes in detail the CNN-BiLSTM-Attention model we propose; the fourth part analyzes and discusses the experimental results; and finally, the fifth part summarizes the research contributions of this paper and looks ahead to future research directions.

## 2. RFI’s Effect on the SNR of the Occultation Satellite Receiver Tracking Signal

### 2.1. Factors Affecting the SNR of an Occultation Satellite Receiver

Various internal and external factors affect the GNSS RO satellite receiver tracking signal’s SNR [35]. Internal factors mainly include the receiving antenna gain, hardware temperature, and system noise inside the receiver [33]. These factors are difficult to correct and have strong randomness. External factors mainly come from natural noise in the signal propagation path, signal interruption due to scintillation, signal interruption, and interference, which can all cause a drop or fluctuation in the SNR. However, the effects of scintillation and signal interruption on interference detection can be effectively eliminated through appropriate algorithms.

### 2.2. Eliminate the Impact of Scintillation and Signal Interruptions on SNR

**Figure 1:**
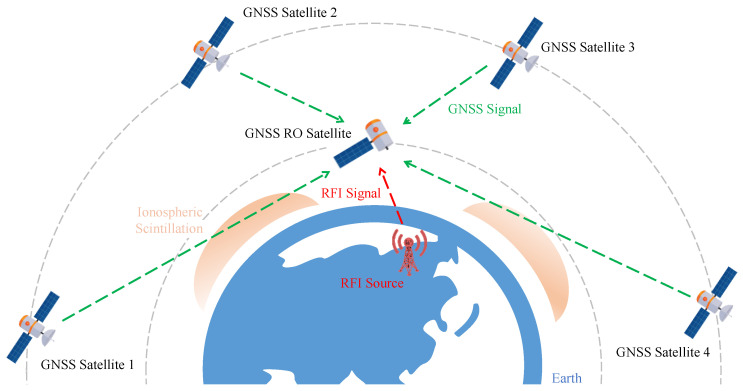
The different signal transmission paths between the RFI source, the GNSS satellites, and the GNSS RO satellites (not to scale).

Most GNSS satellites are medium-Earth orbit (MEO) satellites. GNSS RO satellites usually receive signals with two to three antennas. The upward antenna receives GNSS signals for orbit determination, the forward or backward antenna is used for radio occultation missions, and the downward antenna receives other signals, such as reflections from the Earth [14,33,36]. Figure 1 compares the signal transmission paths between the RFI source, GNSS satellites, and GNSS RO satellites. Since the elevation angles of the signals from different GNSS satellites are different, and so are the propagation paths, it is impossible for all GNSS signals to pass through the same ionospheric scintillation region simultaneously. Signal interruptions caused by an elevation angle that is too low will not co-occur. However, interference signals have full-band characteristics and can simultaneously increase the noise level of all signals, resulting in a simultaneous decrease in the SNR of all signals.

**Figure 2:**
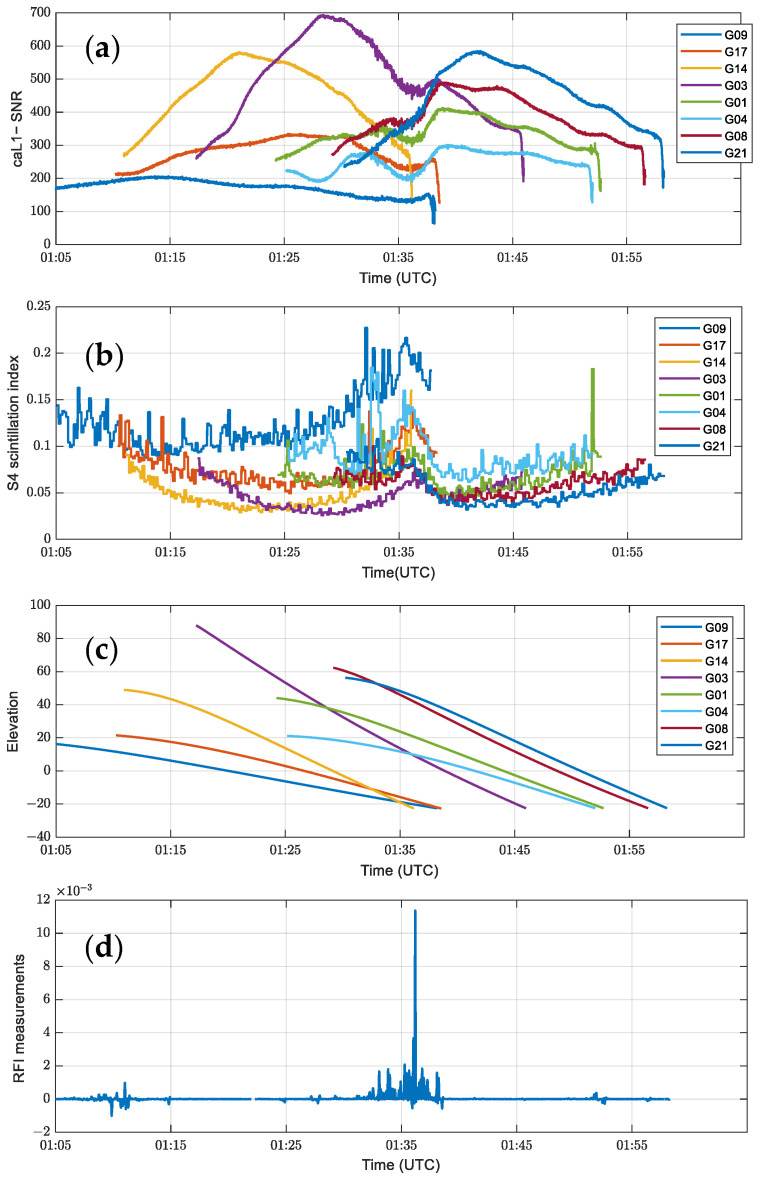
SNR, S4 scintillation index, elevation angle, and RFI measurements for the POD 01 antenna of C2E1 satellite near 1:35 UTC on 1 January 2023: (**a**) SNR sequence of CA code L1 band for different channels. (**b**) S4 scintillation index. (**c**) Elevation angle of the LEO-GPS link. (**d**) Maximum value of RFI measurements in multiple channels.

The sensitivity of the constellation system to interference has been significantly improved due to the reduction in the height of the COSMIC-2 mission, adjustments to the antenna design and orientation, and a higher sensitivity RF front end [37]. Figure 2 shows the overall decline in the SNR during ground interference, and the changes show a high degree of correlation. Researchers have developed algorithms using the SNR as the observation quantity to detect and quantify these ground RFI events [33]. In addition, we note that COSMIC-2 Level 1b Nrt products updated the RFI (Radio Frequency Interference index) measurements in mid-2022.

Figure 2 shows the SNR sequences, S4 scintillation index, and elevation angle of the LEO-GPS link for different channels of the C2E1 satellite POD (Precision Orbit Determination) 01 antenna near 1:35 UTC on 1 January 2023, as well as the maximum of the sequence of RFI measurements for multiple channels. The elevation angle in Figure 2c will lead to signal interruption if it is out of the signal reception range, so the SNR in Figure 2a appears at different times in different channels.

**Figure 3:**
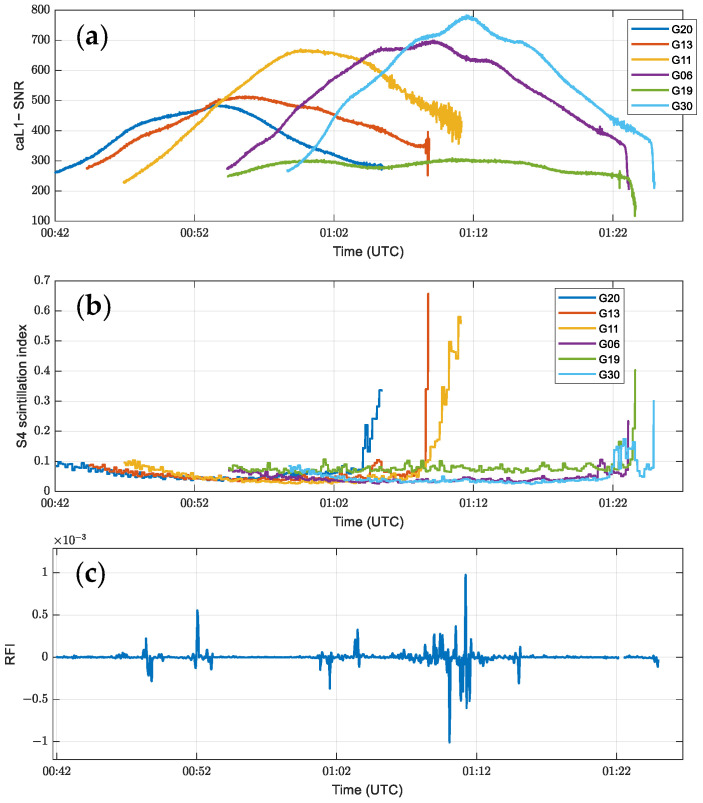
Changes in SNR, S4 scintillation index, and RFI measurements of the C2E1 satellite POD 01 antenna when scintillation occurs on 1 January 2023 near 1:10 UTC. (**a**) SNR sequence of CA code L1 band for different channels. (**b**) S4 scintillation index. (**c**) Maximum value of RFI measurements in multiple channels.

During the period around 1:35 UTC, according to Figure 2b, it can be seen that most of the S4 scintillation indices in the ionosphere are less than 0.2, and no significant scintillation is observed [38]. However, it can be seen from Figure 2d that the RFI measurements are abnormally elevated despite the elevation angle being within the signal reception range. Further analysis reveals that the region of abnormally elevated RFI corresponds to the phenomenon of correlated oscillations and decreases in the SNR. In order to explore the difference between the effects of scintillation and RFI on SNR, Figure 3 shows the variation in SNR in each channel of the C2E1 satellite POD 01 antenna on 1 January 2023 near 1:10 UTC when scintillation occurs.

According to Figure 3b, the S4 scintillation indices of the G13 and G11 channels are more significant than 0.5 near 1:10 UTC, indicating strong scintillation. Corresponding to Figure 3a, the SNRs of the two satellites show significant oscillations and decreases at this time. Meanwhile, the RFI measurements in Figure 3c are maintained at a low level of 10^−3^, and no significant RFI is observed. Thus, unlike the RFI effect, scintillation caused oscillations and decreases in the SNR only for two channels, G13 and G11, without affecting all channels.

Based on the above analysis, the following conclusions can be drawn: The SNR changes in the GNSS RO satellite receiver due to ionospheric scintillation are asynchronous and uncorrelated, and only some GNSS signals are affected. The sudden drop in the SNR due to signal loss caused by the GNSS satellite exceeding the receiver’s signal reception elevation angle is also an uncorrelated phenomenon. In contrast, interference will cause the SNR of all signals to decrease or fluctuate synchronously.

Given these conclusions, the SNR sequence can be band-pass filtered to eliminate the effects of scintillation and signal interruption, and after normalizing the filtered sequence, the cross-correlation value of each window can be calculated using a sliding window. Finally, the interference events can be detected by setting a threshold. The specific algorithm flow is calculated as follows:

(1) Definition of Variables and Interference Model.

Let the signal power be denoted as $P_{s}$, the system noise power as $P_{n}$, and the interference power as $P_{i}$. The interference power is modeled as a combination of a constant term $P_{i,0}$ and a time-varying term $P_{i,\mathrm{var}}$:

(1) $$P_{i}=P_{i,0}+P_{i,\mathrm{var}}$$

The signal-to-noise-and-interference ratio (SNIR) is then expressed as

(2) $$\mathrm{SNIR}=\frac{P_{s}}{P_{n}+P_{i}}=\frac{P_{s}}{P_{n}+P_{i,0}+P_{i,\mathrm{var}}}$$

(2) Band-pass Filtering and the Impact of Time-Varying Terms.

Over a short time interval, $P_{s}$ and $P_{n}$ can be approximated as constants, while $P_{i,\mathrm{var}}$ is a rapidly varying stochastic component. Considering the influence of band-pass filtering on the SNIR, the variance of the filtered SNIR is given by

(3) $$\mathrm{Var}(\mathrm{SNIR})={\left(\frac{𝜕\mathrm{SNIR}}{𝜕P_{i}}\right)}^{2}\mathrm{Var}(P_{i,\mathrm{var}})$$

Taking the partial derivative of the SNIR with respect to $P_{i}$, we have

(4) $$\frac{𝜕\mathrm{SNIR}}{𝜕P_{i}}=-\frac{P_{s}}{{\left(P_{n}+P_{i,0}\right)}^{2}}$$

Substituting this derivative into the variance equation yields

(5) $$\mathrm{Var}(\mathrm{SNIR})={\left(-\frac{P_{s}}{{\left(P_{n}+P_{i,0}\right)}^{2}}\right)}^{2}\mathrm{Var}(P_{i,\mathrm{var}})=\frac{P_{s}^{2}}{{\left(P_{n}+P_{i,0}\right)}^{4}}\mathrm{Var}(P_{i,\mathrm{var}})$$

(3) Normalized Signal Fluctuations.

To facilitate analysis and comparison, we normalize the filtered SNIR fluctuations. The normalized fluctuation is defined as

(6) $${\Delta \mathrm{SNIR}}_{\mathrm{norm}}=\frac{\Delta \mathrm{SNIR}}{\mathrm{SNIR}}=\frac{\frac{P_{s}}{{\left(P_{n}+P_{i,0}\right)}^{2}}}{\frac{P_{s}}{P_{n}+P_{i}}}P_{i,\mathrm{var}}$$

Simplifying this expression, we obtain

(7) $${\Delta \mathrm{SNIR}}_{\mathrm{norm}}=\frac{P_{n}+P_{i}}{{\left(P_{n}+P_{i,0}\right)}^{2}}P_{i,\mathrm{var}}$$

This formulation captures the nonlinear relationship between interference power fluctuations and normalized signal fluctuations while decoupling from the signal power $P_{s}$.

(4) Voltage-Based Signal-to-Noise Ratio.

The SNR reported by the COSMIC-2 receiver TriG is expressed as the voltage signal-to-noise ratio ${\mathrm{SNR}}_{v}$ [39], and its relationship with the power-based signal-to-noise ratio ${\mathrm{SNR}}_{p}$ is as follows:

(8) $${\mathrm{SNR}}_{v}\propto \sqrt{{\mathrm{SNR}}_{p}}$$

Thus, the normalized voltage SNR fluctuation can be expressed as

(9) $${\Delta \mathrm{SNR}}_{v,\mathrm{norm}}=\frac{\sqrt{P_{n}+P_{i}}}{P_{n}+P_{i,0}}\sqrt{P_{i,\mathrm{var}}}$$

(5) Cross-Correlation Across Channels.

To assess the impact of RFI, we compute the cross-correlation of normalized SNR fluctuations across multiple channels. Denoting the normalized fluctuation of the *i*-th and *j*-th channels at time *t* as

(10) $$x_{i}(t)={\Delta \mathrm{SNR}}_{v,i,\mathrm{norm}},\;x_{j}(t-\tau )={\Delta \mathrm{SNR}}_{v,j,\mathrm{norm}}$$

The cross-correlation metric is defined as

(11) $$M(t)=\frac{1}{N}\sum_{i=1}^{N}\sum_{j=1}^{N}\sum_{\tau =0}^{T}x_{i}(t)x_{j}(t-\tau )$$

where *N* is the total number of channels, and *T* is the time window length. A significant increase in *M*(*t*) indicates high correlation among signal fluctuations across channels, which is a strong indicator of RFI presence.

**Figure 4:**
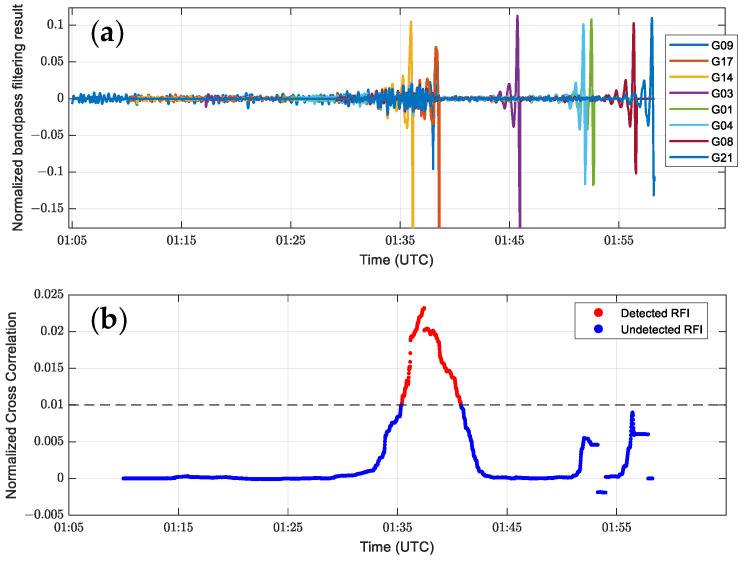
Results of the interference detection algorithm: (**a**) The result of band-pass filtering and normalization of the multi-channel SNR sequence in Figure 2a. (**b**) The calculated cross-correlation sequence after moving window normalization and filtering, as well as interference, can be detected by setting a threshold (set to 0.01 in this example).

**Figure 5:**
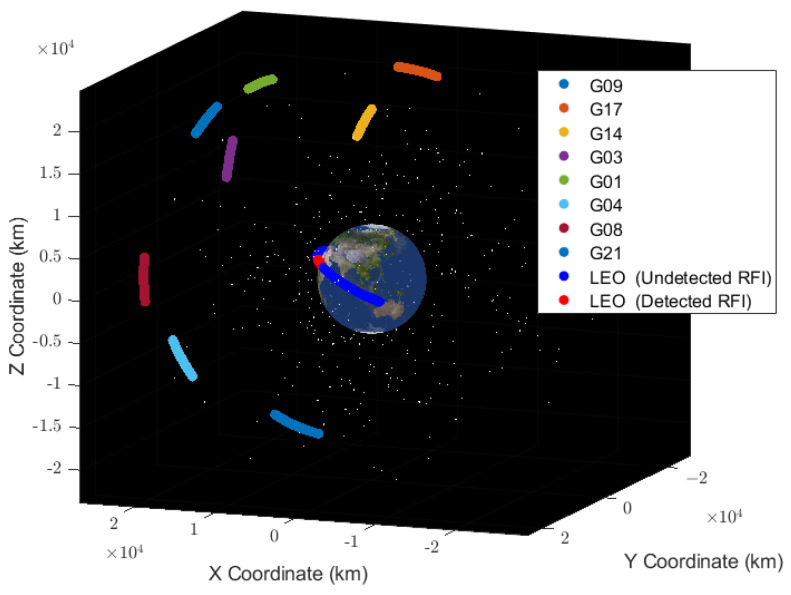
Spatial distribution of the orbits of GPS and COSMIC-2 satellites during the SNR duration in Figure 2a.

Using the RFI detection algorithm on the SNR sequence in Figure 2a, Figure 4 shows the results of the band-pass filtered and normalized multi-channel SNR sequence calculated by Equation (9) and the cross-correlation results of all combinations of the trajectories at the same time as the band-pass filtered and normalized by Equation (10). Figure 5 also shows the distribution of GPS satellites and COSMIC-2 satellites during this period.

Through principal component analysis of the SNR sequence spectrum and repeated experiments, it can be determined that a seventh-order digital Butterworth band-pass filter with upper and lower frequency limits of 0.075 and 0.02 Hz can produce robust detection [33]. However, this method has certain limitations; the filter parameters must be dynamically adjusted according to the specific circumstances of different years and constellation systems. The filtering effect directly affects the accuracy of interference detection. If the filtering effect is not good, interference cannot be effectively detected.

**Figure 6:**
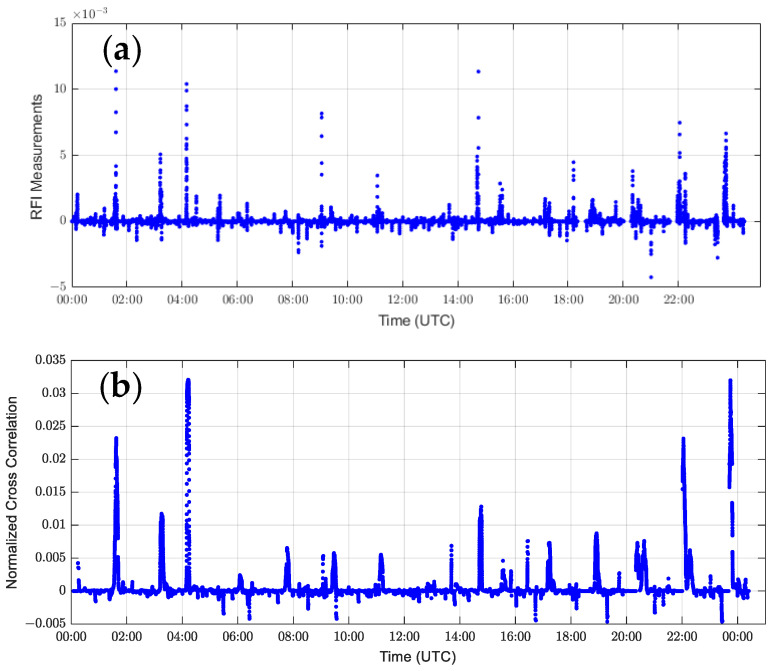
Calculation results of the RFI measurement sequence and cross-correlation sequence output by the C2E1 satellite 01 antenna on January 1, 2023 UTC. The two sequences also show a certain degree of correlation over the day: (**a**) RFI measurement sequence; (**b**) Calculated normalized cross-correlation sequence after band-pass filtering.

In Figure 4a,b, the cross-correlation sequence after band-pass filtering and the RFI measurements value highly match around UTC 1:35, and both show a significant abnormal increase when the interference event occurs. This indicates a strong correlation between interference events and signal characteristics. Figure 6 further shows the simultaneous changing trends of the SNR band-pass filtered cross-correlation sequence and the RFI measurements value on a larger time scale (January 1, 2023 UTC). It can be observed that the moment when the RFI measurements value increases corresponds to the simultaneous increase in the SNR cross-correlation sequence, indicating a clear correlation between the RFI measurements value and the SNR.

These results show that predicting changes in RFI measurements based on the SNR is feasible. By setting an appropriate threshold, RFI values exceeding the threshold can be marked as interference events, enabling automatic interference detection. Based on this idea, multi-channel SNR sequences and LEO orbital coordinate sequences can be used as inputs to deep learning models. A neural network model is constructed to perform time series regression, and the model’s output is the predicted RFI value. Analyzing the predicted RFI value makes it possible to determine whether the system is being interfered with.

## 3. Models

In this paper, the multi-channel SNR and maximum RFI measurements with a rate of 1 Hz generated by POD 01 and 02 antennae of the four satellites C2E1, C2E2, C2E3, and C2E4 of COSMIC-2 in 2023 are selected as the training set, and the corresponding data of the two satellites C2E5 and C2E6 are used as the test set. The 1 Hz multi-channel SNR sequence is used as the model input, and the model output is the maximum value of the 1 Hz multi-channel RFI measurement. The CNN-BiLSTM-Attention model proposed in this paper integrates three deep learning network structures: convolutional neural networks, bidirectional extended short-term memory networks, and attention mechanisms.

### 3.1. CNN

**Figure 7:**
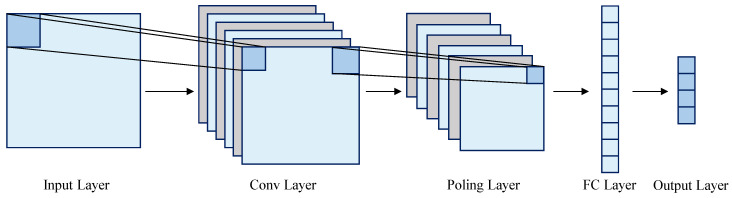
Basic structure of a CNN model.

Convolutional neural networks (CNNs) can use convolution operations to express raw data at a higher level and move abstractly, and they excel in fields such as image processing [40]. Since SNR time series data have local correlations in the presence of interference, CNNs are suitable for processing local features. The basic structure of a CNN is shown in Figure 7 and mainly consists of convolutional and pooling layers [41]. The convolutional layer uses a convolutional kernel to effectively extract the nonlinear local features of RFI data. In contrast, the pooling layer compresses the extracted features and improves the model’s generalization ability.

### 3.2. BiLSTM

**Figure 8:**
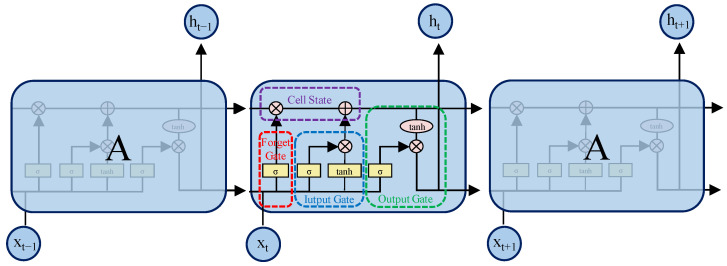
LSTM model schematic.

The extended short-term memory network (LSTM) was proposed by Schmidhuber in 1997 to solve the problem of gradient disappearance in RNN models [42]. Figure 8 shows the internal structure of the LSTM model, which consists of an input gate, a forget gate, and an output gate, to determine the memorization and forgetting of information at each moment. LSTM is widely used in time series prediction [43,44]. Its calculation formula is as follows:

(12) $$f_{t}=\sigma (W_{x_{f}}x_{t}+W_{h_{f}}h_{t-1}+b_{f})$$

(13) $$i_{t}=\sigma (W_{x_{i}}x_{t}+W_{h_{i}}h_{t-1}+b_{i})$$

(14) $${\tilde{C}}_{t}=\tanh(W_{x_{c}}x_{t}+W_{h_{c}}h_{t-1}+b_{c})$$

(15) $$C_{t}=f_{t}⊙C_{t-1}+i_{t}⊙{\tilde{C}}_{t}$$

(16) $$o_{t}=\sigma (W_{x_{o}}x_{t}+W_{h_{o}}h_{t-1}+b_{o})$$

(17) $$h_{t}=o_{t}⊙\tanh(C_{t})$$

Among them, $f_{t}$ is the output of the forget gate, $W_{x_{f}}$, $W_{h_{f}}$ is the weight of the forget gate, $W_{x_{i}}$, $W_{h_{i}}$ is the weight of the input gate, $W_{x_{c}}$, and $W_{h_{o}}$ is the weight of the output gate, $b_{f}$, $b_{i}$, and $b_{o}$ are bias terms, $o_{t}$ is the output of the internal output gate of the neuron, and $h_{t}$ is the output of the neuron at the current time. σ is the Sigmoid activation function.

**Figure 9:**
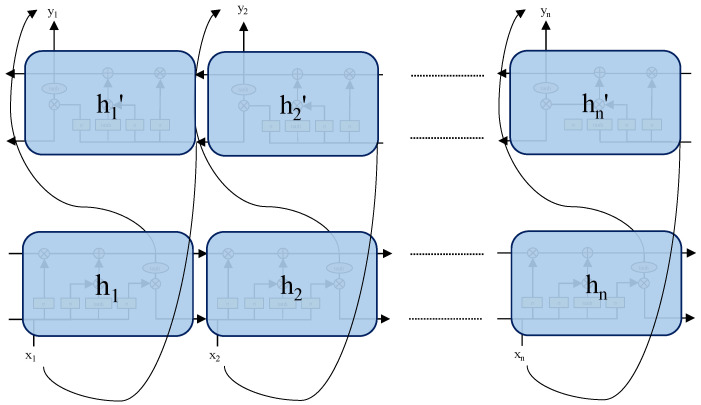
Schematic diagram of the BiLSTM model.

The structure of BiLSTM is shown in Figure 9. Since LSTM only supports unidirectional feature information transmission, BiLSTM uses Equations (18) and (19) to input the sequence forwardly and reversely into the two LSTM networks for feature extraction and then uses Equation (20) to concatenate the output vectors, thereby enhancing the ability to capture contextual information and improving model performance.

(18) $${\vec{h}}_{t}=LSTM(x_{t},{\vec{h}}_{t-1})$$

(19) $${\overset{\leftarrow }{h}}_{t}=\mathrm{LSTM}(x_{t},{\overset{\leftarrow }{h}}_{t-1})$$

(20) $$y_{t}=\vec{W}{\vec{h}}_{t}+\overset{\leftarrow }{W}{\overset{\leftarrow }{h}}_{t}+b_{y}$$

Among them, $x_{t}$ is the input data at t, ${\vec{h}}_{t}$ and ${\overset{\leftarrow }{h}}_{t}$ are the output of the forward LSTM hidden layer, and the output of the backward LSTM hidden layer; $\vec{W}$ and $\overset{\leftarrow }{W}$ are the weights of ${\vec{h}}_{t}$ and ${\overset{\leftarrow }{h}}_{t}$.

### 3.3. Attention Mechanism

This paper introduces an attention mechanism after the BiLSTM layer, which endows the model with the ability to model the dependency between different positions, enabling the model to process the entire input sequence in one step and dynamically assign weights according to the importance of the elements, thereby improving prediction performance by effectively capturing relevant information in the sequence. In the BiLSTM attention mechanism model, the attention module receives the bidirectional temporal features output by the BiLSTM module. It dynamically assigns weights to the features according to the actual situation.

In the attention mechanism module, the similarity between the features and the regression values is first calculated to obtain the attention score:

(21) $$Sim(h_{i},y_{t})=h_{i}\cdot y_{t}$$

where $h_{i}$ is the ith hidden state, $y_{t}$ is the target vector, and $Sim(h_{i},y_{t})$ is the similarity between $h_{i}$ and $y_{t}$.

The attention score is then normalized using the softmax function, which rearranges the raw calculated score into a probability distribution where all element weights and the weight of the critical feature are 1:

(22) $$a_{i}=Softmax(Sim_{i})=\frac{e^{Sim_{i}}}{\sum_{j=1}^{L_{\chi }}e^{Sim_{j}}}$$

Among them, $a_{i}$ is the normalized weight.

The weighted sum of the features is calculated according to the weight coefficient to obtain the weighted feature ${\tilde{H}}_{i}$:

(23) $${\tilde{H}}_{i}=\sum_{i=1}^{L_{x}}a_{i}\cdot h_{i}$$

where ${\tilde{H}}_{i}$ is the weighted representation of the input sequence weighted by the attention mechanism, and $L_{x}$ is the sequence length.

### 3.4. CNN-BiLSTM-Attention

CNN is good at extracting the features of time series data, but it has difficulty dealing with long dependencies. BiLSTM, on the other hand, is effective at learning time series and solves this shortcoming of CNN. Therefore, combining CNN and BiLSTM to form a CNN-BiLSTM model can give full play to their respective advantages and improve prediction accuracy [45]. However, this model may ignore important features when processing high-dimensional inputs and large-scale data, which affects learning ability and prediction accuracy. Introducing an attention mechanism can capture the impact of feature pairs of data at different moments on the predicted value, thereby improving the model’s prediction accuracy [46].

**Figure 10:**
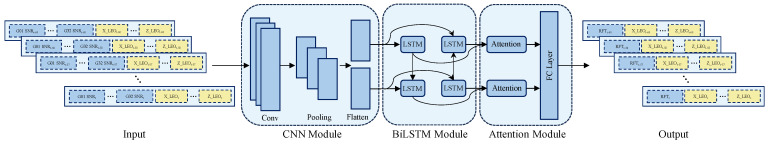
Basic structure of the CNN-BiLSTM-Attention model used in this paper.

**Figure 11:**
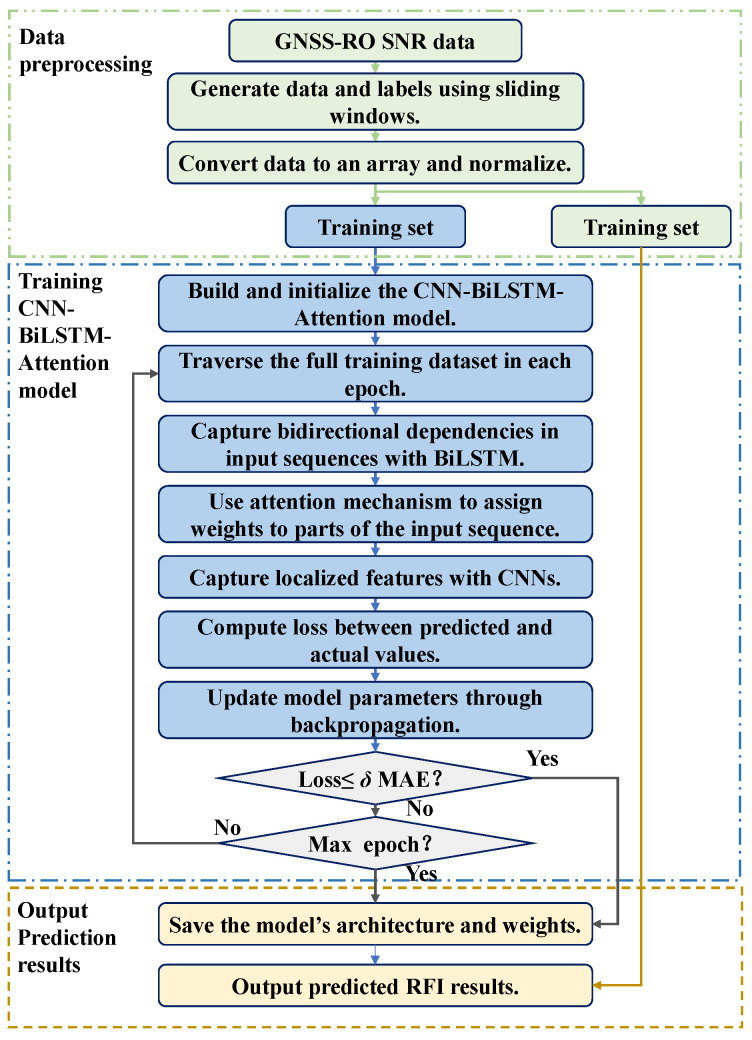
Flowchart of the algorithm.

As shown in Figure 10, the CNN-BiLSTM-Attention model consists of an input layer, a CNN layer, a BiLSTM layer, an Attention layer, and an output layer. In the input layer, the sliding window is set to 60 s, considering that the duration of RFI is mostly between 10 and 50 s. The input data consist of a historical time series of SNR data from 32 GPS satellites and Earth-Centered, Earth-Fixed (ECEF) coordinates of LEO satellites. All SNR values are interpolated to a continuous expected time base, where the time of untracked values is filled with 0. The CNN layer performs feature extraction on the input data and selects essential features, and the pooling layer performs dimensionality reduction. The BiLSTM layer calculates the time series by passing the extracted data through multiple forward and backward LSTM units. The Attention layer calculates the influence of the feature state of the data on the predicted value at each moment. The output layer performs the final calculation on the data output by the Attention layer to obtain the max RFI measurements time series and the ECF coordinates of the LEO satellite within the sliding window. The LEO satellite coordinates are used for subsequent interference locations and do not participate in the training process. The detailed parameter configuration of the model is shown in Table 1, and the flowchart of the algorithm is shown in Figure 11.

### 3.5. Model Evaluation

In this paper, the performance of the proposed deep learning algorithm in predicting RFI measurements is comprehensively evaluated using indicators such as mean absolute error (MAE), root mean square error (RMSE), correlation coefficient (R^2^), and RFI residual (dRFI). Since RFI measurements are mainly concentrated around 0 without interference, traditional evaluation metrics such as RMSE, MAE, and R^2^ are sensitive to outliers and may not fully reflect the model’s effectiveness. For this reason, a logarithmic transformation is introduced to reduce the impact of large values on the evaluation results and make the metrics more sensitive to small fluctuations close to zero. The formula is as follows:


(24) $$RMSE_{Log}=\sqrt{\frac{1}{n}\sum_{i=1}^{n}{\left(\log(y_{i}+C)-\log\left({\hat{y}}_{i}+C\right)\right)}^{2}}$$

(25) $$MAE_{Log}=\frac{1}{n}\sum_{i=1}^{n}|\log(y_{i}+C)-\log({\hat{y}}_{i}+C)|$$

(26) $$R_{Log}^{2}=1-\frac{\sum_{i=1}^{n}{\left(\log(y_{i}+C)-\log({\hat{y}}_{i}+C)\right)}^{2}}{\sum_{i=1}^{n}{\left(\log(y_{i}+C)-\bar{\log(y+C)}\right)}^{2}}$$

(27) $$dRFI=RFI_{True}-RFI_{Pre}$$

Among them, n is the total number of samples, $y_{i}$ is the actual value of sample i, ${\hat{y}}_{i}$ is the predicted value of sample i, and $\bar{\log(y+C)}$ is the logarithmically averaged value of all sample actual values. In order to avoid the meaningless operation of $log\left(0\right)$ when taking the logarithm, a minimal positive number C is added to the calculation process.

## 4. Result and Discussion

### 4.1. Model Performance Analysis

**Figure 12:**
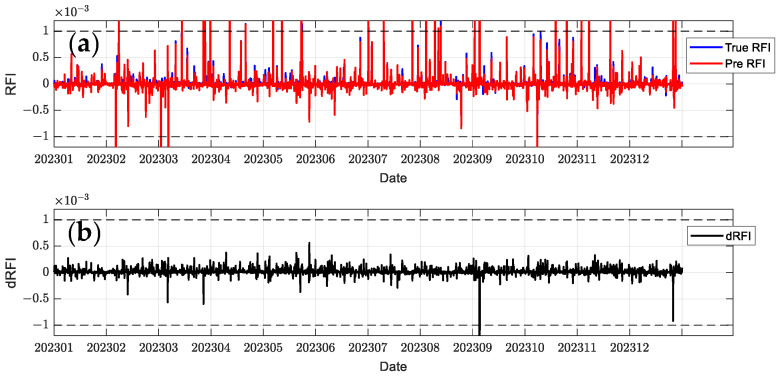
Predicted RFI measurements and dRFI for the training set on the COSMIC-2 C2E1 satellite for the CNN-BiLSTM-Attention model. The dashed line in the figure indicates the interference threshold y = ±0.001, with the time resolution downsampled to 3 h: (**a**) the blue line is the true value of the RFI measurement, and the red line is the prediction result; (**b**) dRFI.

**Figure 13:**
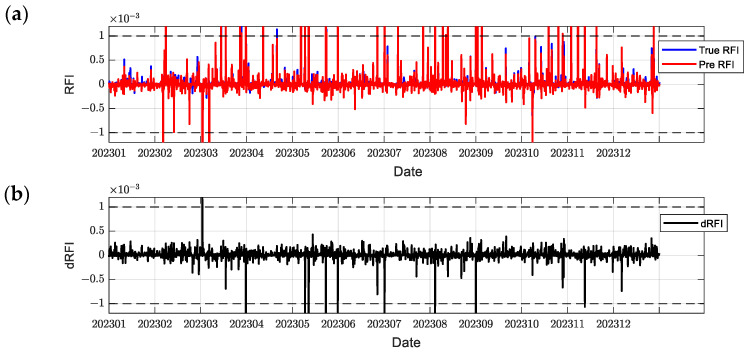
Predicted RFI measurements and dRFI for the training set on the COSMIC-2 C2E1 satellite for the BiLSTM-Attention model. The dashed line in the figure indicates the interference threshold y = ±0.001, with the time resolution downsampled to 3 h: (**a**) the blue line is the true value of the RFI measurement, and the red line is the prediction result; (**b**) dRFI.

**Figure 14:**
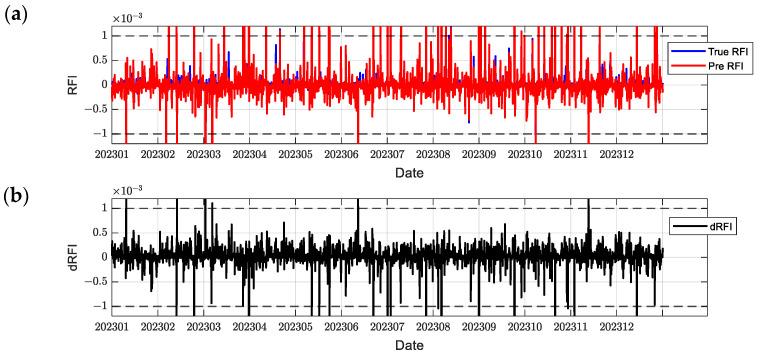
Predicted RFI measurements and dRFI for the training set on the COSMIC-2 C2E1 satellite for the LSTM model. The dashed line in the figure indicates the interference threshold y = ±0.001, with the time resolution downsampled to 3 h: (**a**) the blue line is the true value of the RFI measurement, and the red line is the prediction result; (**b**) dRFI.

**Figure 15:**
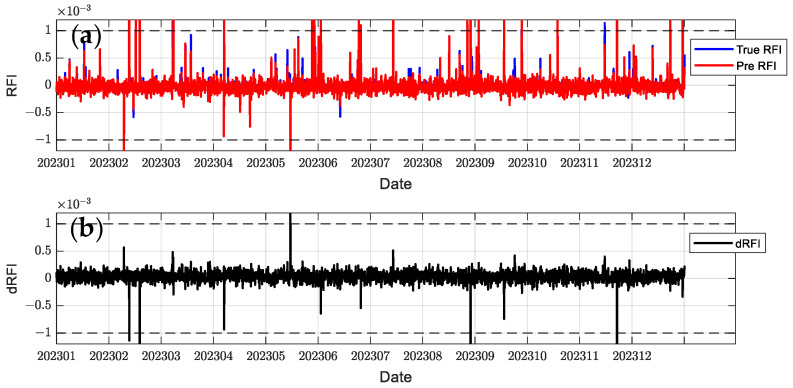
Predicted RFI measurements and dRFI for the test set on the COSMIC-2 C2E1 satellite for the CNN-BiLSTM-Attention model. The dashed line in the figure indicates the interference threshold y = ±0.001, with the time resolution downsampled to 3 h: (**a**) the blue line is the true value of the RFI measurement, and the red line is the prediction result; (**b**) dRFI.

**Figure 16:**
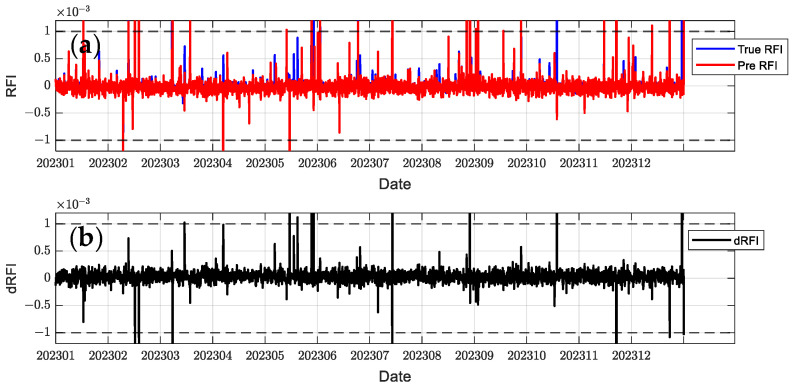
Predicted RFI measurements and dRFI for the test set on the COSMIC-2 C2E1 satellite for the BiLSTM-Attention model. The dashed line in the figure indicates the interference threshold y = ±0.001, with the time resolution downsampled to 3 h: (**a**) the blue line is the true value of the RFI measurement, and the red line is the prediction result; (**b**) dRFI.

**Figure 17:**
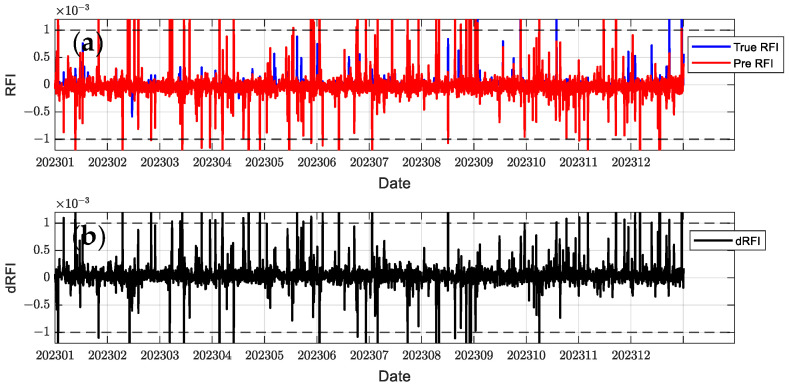
Predicted RFI measurements and dRFI for the test set on the COSMIC-2 C2E1 satellite for the LSTM model. The dashed line in the figure indicates the interference threshold y = ±0.001, with the time resolution downsampled to 3 h: (**a**) the blue line is the true value of the RFI measurement, and the red line is the prediction result; (**b**) dRFI.

In order to comprehensively evaluate the model performance, this paper selects C2E1, C2E2, C2E3, and C2E4 of the COSMIC-2 satellite in 2023 as the training set and C2E5 and C2E6 as the test set. It compares the performance of the BiLSTM-Attention and LSTM models. The parameters of all models were determined using a grid search method to find the optimal configuration. Figure 12, Figure 13, Figure 14, Figure 15, Figure 16 and Figure 17 show the RFI measurements prediction results of different models on the training and test sets. Figure 12, Figure 13 and Figure 14 show the time series plot of the RFI measurements prediction values, actual values, and residuals of the C2E1 satellite data in 2023, and Figure 15, Figure 16 and Figure 17 show the prediction results of the C2E6 satellite for the same period. All time series data have been downsampled to a resolution of 3 h. The blue line shows the actual RFI measurement, the red line shows the RFI measurement predicted by the model, and the black line shows the RFI residual value calculated by Equation (27).

Since the threshold for RFI measurement is 0.001, when dRFI exceeds this value, it will significantly affect the accuracy of interference detection. As shown in Figure 12, the CNN-BiLSTM-Attention model only has one time when dRFI exceeds the substantial interference threshold in the data for the entire year, and the remaining errors are within an acceptable range. Although this model has some deviation in the area close to 0, it can still better capture changes in high RFI values. In contrast, the BiLSTM-Attention model in Figure 13 has a significantly higher number of dRFI occurrences, and most of the high-value dRFI is negative, indicating a bias in high-value predictions. The LSTM model in Figure 14 has a significantly higher number of dRFI occurrences than the previous two and frequently exceeds the threshold. The overall prediction results are high, which may adversely affect interference detection.

In the test set (Figure 15, Figure 16 and Figure 17), the performance of all models decreased, but the CNN-BiLSTM-Attention model still performed best. Statistical results show that CNN-BiLSTM-Attention is superior to other models in terms of RMSE, MAE, and R^2^ in logarithmic form, as shown in Table 2.

Even though most of the points in the dataset are clustered around 0, the CNN-BiLSTM-Attention model still outperforms the other models in terms of evaluation metrics and, in particular, performs better in terms of RMSE (1.0185), MAE (1.8567), and R^2^ (0.9693), demonstrating its high accuracy in RFI measurements prediction tasks.

### 4.2. Global RFI Source Location Results

Grid the global latitude and longitude based on a spatial resolution of 2.5° (latitude) × 5° (longitude), count the grid locations where the RFI measurement is more significant than 0.001 and generate a global interference situation map. Due to the orbital inclination of the COSMIC-2 satellite of about 70 degrees, affected by the Earth’s rotation, the satellite’s observation range is mainly concentrated in the equatorial and low latitude regions, and its trajectory is mainly distributed between 24° north and south latitude. The limited coverage leads to a higher revisit frequency in these areas, resulting in a higher spatiotemporal resolution of the interference situation distribution. This paper plots the actual measured RFI values, as well as the predicted RFI values from the CNN-BiLSTM-Attention, BiLSTM-Attention, and LSTM models, and the global interference situation map in 2023 using the method of calculating the cross-correlation with band-pass filtering in the case of superposition of the 01 and 02 antennas of the six satellites.

**Figure 18:**
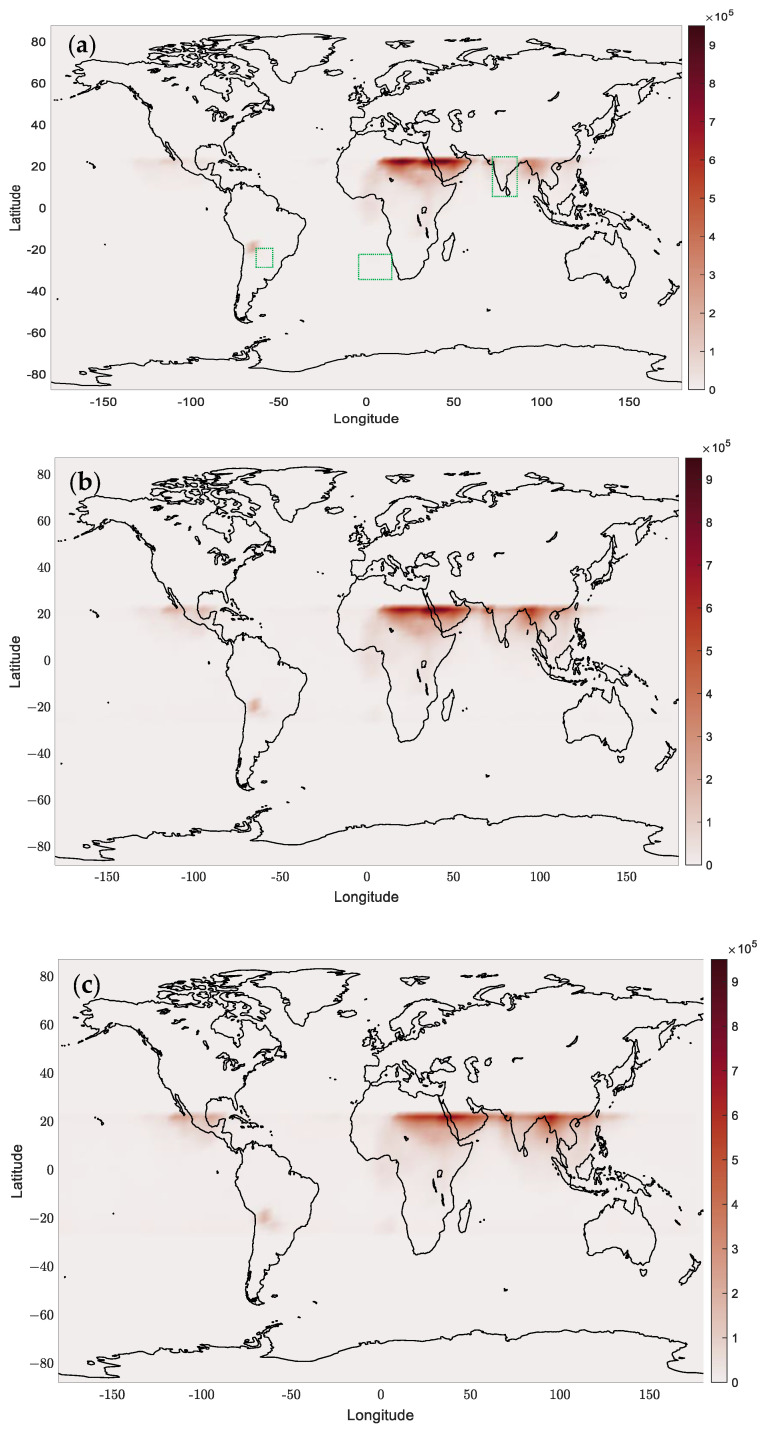

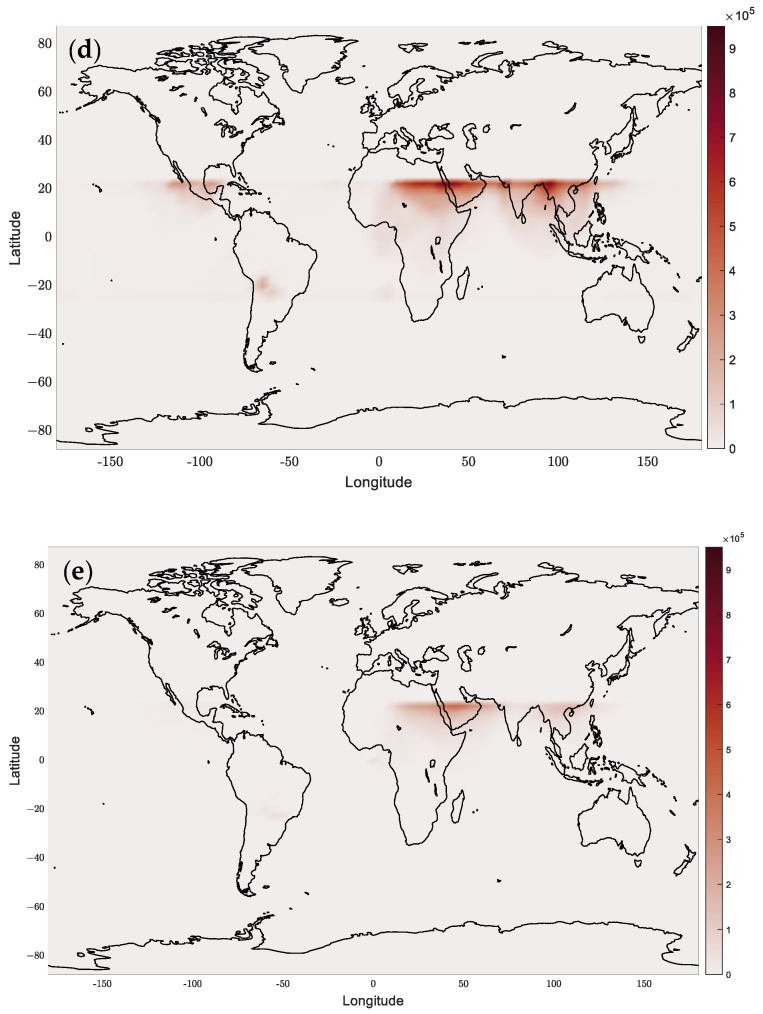
Global RFI situation map of the six COSMIC-2 satellites’ 01 and 02 antenna superposition cases using the four methods mentioned in this paper and the actual measured values: (**a**) real measured values, and the three green dotted rectangles inside the marker are the primary sources of prediction error for various algorithms (**b**) CNN-BiLSTM-Attention, (**c**) BiLSTM-Attention, (**d**) LSTM, (**e**) normalized cross-correlation method with band-pass filtering.

As shown in Figure 18, global GNSS interference is mainly concentrated in the Middle East, which has become the hardest-hit area due to the region’s complex geopolitical environment and frequent activities of interference sources. The spatial distribution of interference is reflected in the different models in Figure 18, where Figure 18a shows the accurate interference distribution. In contrast, Figure 18b–d show the interference situation predicted by three different deep learning models, and Figure 18e shows the interference distribution calculated by the band-pass filtering normalized cross-correlation method. All models can reflect the interference hotspots in the Middle East, indicating that these models have a high degree of accuracy in capturing vital interference areas. In addition, frequent interference sources have also been observed in four models along the Pakistan–India border and in the Myanmar region. The GPSJAM website (https://gpsjam.org/) (accessed on 26 November 2024) can generate daily updated RFI maps using ADS-B data from civil passenger aircraft. Comparison with the website’s map shows that there was indeed long-term interference in the regions mentioned above in 2023.

However, there is almost no interference signal in the Indian region, as shown in Figure 18a. However, in the prediction results of the deep learning method in Figure 18b–d, there are some false alarms, especially in Figure 18c,d, where there is also a slight false alarm in the border area of 24° north and south latitude. These false alarms may be related to the prediction uncertainty of the model in the marginal areas, indicating that the model may still over-predict in non-interference areas, leading to misjudgments.

It is worth noting that the global interference situation map of the CNN-BiLSTM-Attention model in Figure 18b has a high degree of agreement with the actual interference map (Figure 18a). The number of false alarms is relatively small, especially in the Middle East and other high-interference areas, where the prediction results are closer to the actual distribution. In addition, this model rarely produces false alarms in the border areas at 24° north and south latitudes, indicating that this model has better prediction capabilities than other models in high-latitude border areas. In contrast, the BiLSTM-Attention and LSTM models in Figure 18c,d, although able to capture a wide range of interference situations, have obvious false alarms in some marginal areas, and the predicted global interference distribution is not accurate enough. Figure 18e sets an excessively high threshold (0.01) to eliminate the geometric effects of satellite orbits and environmental effects such as ionospheric scintillation, resulting in many missed detections.

Overall, the CNN-BiLSTM-Attention model performed better in global interference detection, accurately locating interference hotspots and effectively reducing false alarms in marginal areas, demonstrating its potential for GNSS interference detection and location in complex environments.

## 5. Conclusions

This paper applies the CNN-BiLSTM-Attention neural network to the historical time series of the SNR parameters of multi-channel GNSS signals to predict RFI measurement values accurately. A global interference situation map is constructed based on these prediction results. In the data test of the C2E5 and C2E6 satellites, the correlation coefficient between the predicted value and the actual value reached 0.9693, and the prediction error was small, with RMSE and MAE of 1.0185 and 1.8567, respectively, which verified the superior performance of the model in the RFI prediction task. Compared with the BiLSTM-Attention and LSTM models, this model performs better regarding time series residuals, RMSE, MAE, and correlation. In addition, by setting an RFI threshold and combining it with the global latitude and longitude gridding technology of 2.5° (latitude) × 5° (longitude), a global interference situation map closer to the actual situation is constructed.

Current research focuses mainly on the L1 CA GPS signal received by the POD antenna of the COSMIC-2 occultation satellite to achieve interference detection. Future research will further study other GNSS RO satellites, such as occultation satellites, explore the impact of interference on other frequency signals, and combine the amplitude and dynamic characteristics of signal attenuation to detect jamming suppression and spoofing further. This will help us to understand the nature and mechanism of GNSS interference fully.

## Figures and Tables

**Table 1:** Model’s detailed parameter configuration.

Model Parameter	Network
Batch Size: 64	BiLSTM: units = 150
Learning Rate: 0.001	Activation: ‘relu’
Epoch: 100	Attention Dropout: 0.1
Loss Function: MAE	Optimizer: Adam

**Table 2:** RMSE, MAE, R^2^ (logarithmic form) for the three models.

Deep Learning Model	RMSE	MAE	R^2^
CNN-BiLSTM-Attention	1.0185	1.8567	0.9693
BiLSTM-Attention	1.4960	2.1280	0.9203
LSTM	2.0498	2.9194	0.7827

## Data Availability

The original data presented in this study are openly available in UCAR at https://data.cosmic.ucar.edu/gnss-ro/cosmic2/ (accessed on 27 November 2024).
